# INTEREST GROUP SESSION—AGE-FRIENDLY UNIVERSITY (AFU): AGE-FRIENDLY UNIVERSITY CAMPUSES: PUTTING PRINCIPLES INTO PRACTICE

**DOI:** 10.1093/geroni/igz038.1250

**Published:** 2019-11-08

**Authors:** Joann M Montepare, Kimberly Farah, Kenneth F Ferraro

**Affiliations:** 1 Lasell College, Newton, Massachusetts, United States; 3 Purdue University, West Lafayette, Indiana, United States

## Abstract

The pioneering Age-Friendly University (AFU) initiative, endorsed in 2016 by GSA’s Academy for Gerontology in Higher Education (AGHE), calls for institutions of higher education to respond to shifting demographics and the needs of our aging populations through more age-friendly programs, practices, and partnerships. Over 45 institutions in the United States, Canada, European countries, and beyond have joined the network and adopted the 10 AFU guiding principles. This symposium will feature leaders at AFU campuses who will discuss why their institution joined the initiative, their age-friendly campus vision, and how they are putting AFU principles into practice. AFU Washington University St. Louis leaders will describe efforts to increase age-diversity on their campus, especially through professional studies programs that support personal and career development in the second half of life. AFU University of Southern California leaders will discuss new age-friendly efforts entailing intergenerational exchange, best practices of age-friendly programming for retired employees and alumni, and emerging connections to local community aging initiatives. AFU Eastern Michigan University leaders will round out the presentation with an overview of accomplishments of their campus-wide steering committee and its age-friendly evaluation research that informed the needs and interests of older learners pursuing second careers, as well as how to actively engage emeritus faculty and staff as a retired community on campus. The discussant will provide integrating comments and age-friendly suggestions for putting AFU principles into practice.

